# Supplementary material for: Stochastic dynamics of a few sodium atoms in presence of a cold potassium cloud
Source: Sci Rep. 2022 Feb 14;12:2422. doi: 10.1038/s41598-022-05778-8 (PMC8844084; doi:10.1038/s41598-022-05778-8)
Supplement: Supplementary file 1 — Supplementary Information. [file 41598_2022_5778_MOESM1_ESM.pdf]

Supplementary Information for: Stochastic dynamics of a few sodium atoms in presence of a cold potassium cloud

Rohit Prasad Bhatt[1,*], Jan Kilinc[1], Lilo Höcker[1], and Fred Jendrzejewski[1,*]

[1] Universität Heidelberg, Kirchhoff-Institut für Physik, Im Neuenheimer Feld 227, 69120 Heidelberg, Germany
[*]rohit.prasad@kip.uni-heidelberg.de, fnj@kip.uni-heidelberg.de

Simulation of the counting dynamics

We model the microscopic dynamics of the atom counts $\tilde{N}_{\mathrm{at}}(t)$ by a statistical process whose macroscopic parameters are the loading rate Γ_{load} and loss rate Γ_{loss}. For short enough time steps dt the loading of a single atom is described by a Bernoulli trial $\mathcal{B}[1, p_{\mathrm{load}}]$, where $p_{\mathrm{load}} = \Gamma_{\mathrm{load}} dt$. The loss of single atom is described by a binomial process $\mathcal{B}[\tilde{N}_{\mathrm{at}}(t-dt), p_{\mathrm{loss}} = \Gamma_{\mathrm{loss}} dt]$. The change of atom number at time t is then given by

$$\Delta\tilde{N}(t) = \mathcal{B}[1, p_{\mathrm{load}}] - \mathcal{B}[\tilde{N}_{\mathrm{at}}(t-dt), p_{\mathrm{loss}}] \quad (1)$$

Additional interactions between atoms can be modelled microscopically by another binomial process $\mathcal{B}[\tilde{N}_{\mathrm{pairs}}(t-dt), p_2]$, where $\tilde{N}_{\mathrm{pairs}} = \frac{(\tilde{N}_{\mathrm{at}}-1)\tilde{N}_{\mathrm{at}}}{2}$ and p_2 is the loss probability of a pair. However, we neglected this process in our analysis, as all our observations are compatible with $p_2 = 0$.

The atom number at time $t = k \cdot dt$ is then given by

$$\tilde{N}_{\mathrm{at}}(t) = \sum_{k=1}^{t/dt} \Delta\tilde{N}(k \cdot dt)\,, \quad (2)$$

with $\tilde{N}_{\mathrm{at}}(0) = 0$ and $k \in \mathbb{N}$. From (2) we arrive at the collected fluorescence signal through

$$\tilde{N}_{\mathrm{ph}}(t) = \eta\,\gamma_{\mathrm{ph}}\,dt\,\tilde{N}_{\mathrm{at}}(t)\,, \quad (3)$$

where η is the overall photon collection and conversion efficiency, and γ_{ph} the photon scattering rate.

While imaging the atoms in a magneto-optical trap (MOT), the atom fluorescence signal is integrated on a camera over an exposure time τ as visualized in Fig. 1 A. The camera counts $N_{\mathrm{c}}(m)$ of image $m \in \{0, 1, 2...\}$ are then expressed as

$$N_{\mathrm{c}}(m) = \sum_{k=1}^{\tau/dt} \tilde{N}_{\mathrm{ph}}(m \cdot \tau + k \cdot dt) \quad (4)$$

$$= C N_{\mathrm{at}}(m) \quad (5)$$

The terms of equation (5) are the calibration factor $C = \eta\gamma_{\mathrm{ph}}\tau$ and the observed atom number N_{at} from the main text. By repeating the sum in equation (4) for each image m we can simulate the experimental time traces. A typical result of the method is shown in Fig. 1 A.

The single atom counting regime is characterized by average atom number changes $\langle\Delta\tilde{N}\rangle \ll 1$ during the exposure time τ. We can then set $dt = \tau$ and experimentally observe the microscopic dynamics as $N_{\mathrm{at}}(m) = \tilde{N}_{\mathrm{at}}(m \cdot \tau)$. This is the experimentally realized case for sodium.

In the case of large atom numbers and fast dynamics, as observed for potassium in the main

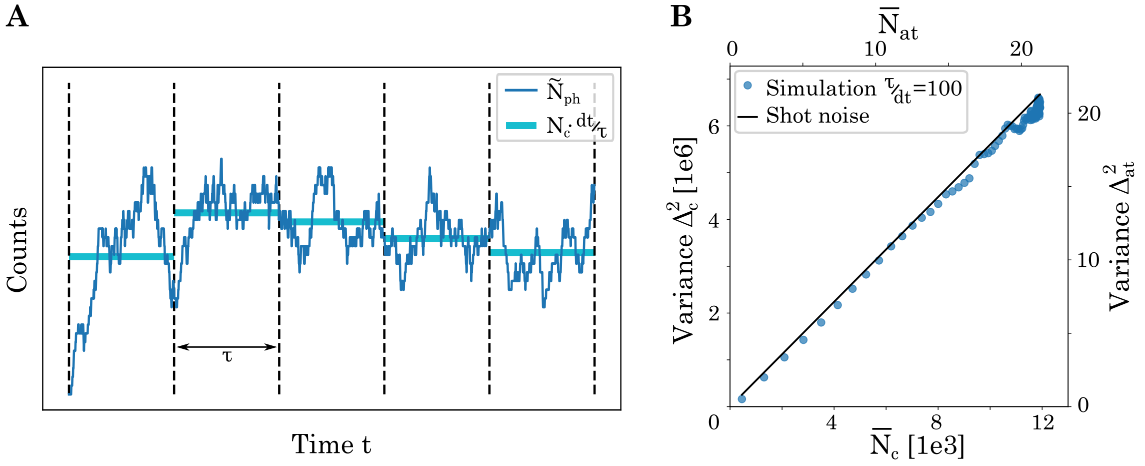

Figure 1: Simulation of the imaging statistics. **A:** The loading and loss processes are characterized by the loading rate Γ_{load} and loss rate Γ_{loss} respectively, while the imaging happens over an exposure time τ. **B:** Dependence of variance on mean camera counts. The shot noise line has a slope of $C = \eta\gamma_{\mathrm{ph}}\tau$.

text, we observe a linear dependence of variance of camera counts on mean camera counts with the slope C. The extracted C from a linear fit agrees well with its value used for generating the time traces (i.e. $C = \eta\gamma_{\mathrm{ph}}\tau$) as shown in Fig. 1 B. This allows us to estimate the calibration factor reliably as discussed in the main text.

The simulation code related to the supplementary information is openly available at the following URL/DOI: `https://doi.org/10.11588/data/HRCX1P`.
